# Influence of salinity on the biometric traits of striped catfish (*Pangasianodon hypophthalmus*) and barley (*Hordeum vulgare*) cultivated under an integrated aquaculture-agriculture system

**DOI:** 10.1186/s12870-023-04422-5

**Published:** 2023-09-09

**Authors:** Muziri Mugwanya, Fahad Kimera, Khaled Madkour, Mahmoud A. O. Dawood, Hani Sewilam

**Affiliations:** 1 Center for Applied Research On the Environment and Sustainability (CARES), School of Science and Engineering, The American University in Cairo, AUC Avenue, P.O. Box 74, New Cairo, 11835 Egypt; 2 Animal Production Department, Faculty of Agriculture, Kafrelsheikh University, Kafr El-Sheikh, Egypt; 3 UNESCO Chair in Hydrological Changes and Water Resources Management, RWTH Aachen University, Aachen, Germany

**Keywords:** Integrated aquaculture-agriculture system, Salinity, Barley, Forage quality, Brackish water, Catfish

## Abstract

**Background:**

Soil salinity, soil infertility, and freshwater scarcity are among the major constraints affecting agricultural ecosystems in arid and semi-arid regions of the world. Hence, there is a need to look for salt-tolerant crops and fish that can be successfully cultivated and reared respectively in such harsh environments. The implementation of biosaline integrated aquaculture-agriculture systems (IAAS) utilizing both salt-tolerant crops and fish could improve food and feed production in arid and semi-arid regions. This study, therefore, investigated the influence of salinity on the biometric traits of striped catfish (*Pangasianodon hypophthalmus*) and barley (*Hordeum vulgare*) under an IAAS.

**Method:**

The experiment followed a randomized completely block design of three salinity treatments with three replicates namely, T0: Control (freshwater mixed with chemical fertilizers), T1: 5,000 ppm, T2: 10,000 ppm, and T3: 15,000 ppm.

**Results:**

Irrigating barley with saline aquaculture wastewater at different salinities (5,000 ppm, 10,000 ppm, and 15,000 ppm) did not significantly affect the agro-morphological parameters (internode number per plant, stalk diameter, leaf number per plant, leaf area index, and leaf chlorophyll content (SPAD)) of the plants at 90 days after sowing. Moreover, the forage yield and forage quality in terms of fiber fraction, nutrient composition, and in vitro digestibility of the forage biomass were not severely affected by high salinity compared to the control (freshwater and inorganic fertilizers). Our results also showed that rearing striped catfish in saline water not exceeding 10,000 ppm did not negatively impact the growth performance (final weight, body weight gain, feed conversion ratio, specific growth rate, condition factor, and survival) and the health status of the fish.

**Conclusions:**

The integration of striped catfish and barley production in water salinities below 15,000 ppm could be a feasible alternative in safeguarding food and feed security in regions affected by soil salinity, soil infertility, and freshwater scarcity. Moreover, the salinity regime of 5,000 ppm could bring higher economic gains to farmers regarding higher crop yields (fish and forage yield).

## Background

Soil salinity, soil infertility, and freshwater scarcity are among the major constraints affecting agricultural ecosystems in arid and semi-arid regions of the world [1–5]. The United Nations Food and Agriculture Organization has estimated that more than 955 million hectares (mha) of land are salt-affected, and ~ 77 mha are attributed to secondary salinization [6]. Soil salinization disrupts the root-soil interactions due to increased salt accumulation in the root zone which changes the soil pH leading to the unavailability of certain nutrients for plant uptake [7, 8]. As such, there is a decline in crop productivity and yield of salt-sensitive plants. On the other hand, freshwater scarcity is another major problem that is globally faced by livestock farmers in arid and semi-arid regions as a consequence of climate change [9–11]. This has accelerated land degradation and loss of biodiversity due to the utilization of saline underground water for irrigation, hence resulting in low crop yields of salt-sensitive forages needed for animal nutrition [12]. In the same regard, the production of alternative protein sources such as fish in inland systems of marginal areas has also been constrained due to limited freshwater resources [13] and hence, further deepening hunger in vulnerable communities. Therefore, to improve both food and feed production in such marginal areas, there is a need to implement scientifically practical and low-cost technologies that make use of on-farm synergies of biosaline crop and fish farming amidst limited natural resources.

Inland saline aquaculture which refers to land-based aquaculture using saline/brackish underground water has provided opportunities to diversify food production as well as improve food security in regions affected by freshwater scarcity [14–16]. Freshwater fish species such as Nile tilapia (*Oreochromis niloticus*), African catfish (*Clarias gariepinus*), and striped catfish (*Pangasianodon hypophthalmus*) are among the most commonly studied species for inland saline aquaculture due to their tolerance to salinity stress [17–22]. For instance, El-Leithy et al. [23] investigated the influence of different salinity concentrations (6000 ppm, 16,000 ppm, and 20,000 ppm) on the growth and immune response of Nile tilapia (*O. niloticus*) and observed better growth performance (body weight gain) and immune response (higher expression of ion regulated genes (*Na*^+^*/K*^+^*-ATPase α1-b*), stress-related genes (*GST, HSP27*, and *HSP70*) of the gills, inflammatory-related genes (*IL-1β* and *IL8*) and immune-related genes (*TLR*) in the liver tissue) of fish reared at 16,000 ppm. At 20,000 ppm, increased mortality and expression of kidney-immune-related genes were noted thus indicating that *O. niloticus* cannot survive well at such water salinities. In another study, Thomas et al. [24] investigated the growth response of *O. niloticus* and spinach (*Spinacia oleracea*) in saline aquaponics system and observed better growth and survival of both fish and plants at 9,000 ppm. For catfish, Zidan et al. [21] observed that rearing *C. gariepinus* in water salinities reaching up to 12,000 ppm did not severely affect the growth performance and immune response in fish compared to those reared at 16,000 and 20, 000 ppm. Kumar et al. [25] conducted a trial to elucidate the salinity tolerance levels (0, 5000, 10,000, 15,000, 20,000 and 25,000 ppm) of *P. hypophthalmus* and observed increased blood urea, reduction in total serum proteins, and poor survival in fish reared at salinities exceeding 15,000 ppm. Using RNA-Seq approach technology, Nguyen et al. [26] observed a differential expression of genes in fish reared at 15,000 ppm, most of which were related to salinity tolerance.

Although inland saline aquaculture presents opportunities for improved food security in marginal areas, management of nutrient-rich aquaculture wastewater is a challenge and its poor disposal could lead to environmental pollution. The integration of aquaculture with agriculture is a feasible alternative for the sustainable utilization of nutrient-rich aquaculture wastewater that would otherwise cause damage to environmental ecosystems [27].

Biosaline integrated aquaculture-agriculture systems (IAAS) have not only proved to be anticipatory actions in safeguarding both food and feed security in marginal areas but also a sustainable source of income from different system components [28, 29]. In these systems, the wastes from one system that would otherwise cause environmental pollution when poorly disposed of are used as an input to another system hence promoting efficient and sustainable utilization of resources (i.e. land and nutrient-rich wastewater) for increased productivity [29–31]. In other words, the aquaculture nutrient-rich wastewater from the fish production units is channeled to the grow beds to be utilized as both a water and nutrient source by the cultivated plants hence increasing yield per unit area of production. For instance, Kimera et al. [30] investigated the influence of aquaculture wastewater on the growth, yield, and essential oil composition of *Origanum majorana* and observed improved growth and biomass yield of the crop irrigated with aquaculture effluents in the first cut. In another study, Tasung et al. [29] reported improved biomass yield in *Salicornia brachiata* Roxb irrigated with aquaculture wastewater compared to seawater. Likewise, Guimaraes et al. [31] investigated the effect of irrigating saline aquaculture wastewater on the productivity of forage sorghum varieties under semi-arid conditions and observed a 25% increase in yield of sorghum irrigated with saline aquaculture wastewater with a 15% leaching fraction. The positive results of the aforementioned studies are attributed to the high concentrations of organic matter, nitrogen, and phosphorus in the aquaculture wastewater all of which improve plant growth and yields. It is imperative to note that organic matter not only improves the soil structure but also lowers soil salinity hence improving plant growth [32–34]. Furthermore, previous studies have shown that aquaculture wastewater contains plant growth-promoting bacteria (PGPB) that stimulate plant tolerance against abiotic stress [35, 36].

To the best of our knowledge, no comparative study has so far been conducted to investigate the growth, yield, and forage quality response of barley (*Hordeum vulgare*) cultivated under an IAAS in comparison to chemical fertilizers.

Barley is one of the most important cereal crops around the world but unlike other economically important cereals like wheat, barley is considered to be relatively salt tolerant [37, 38]. It is worth noting that barley is one of the most commonly studied model crops on the inheritance and mechanisms of salinity tolerance due to its ability to tolerate salinity levels reaching up to 250 mM NaCl (equivalent to 40% sea water) [39, 40]. In the same context, striped catfish (*P. hypophthalmus*) is a freshwater finfish species that has been reported to have tolerance to abiotic stress such as salinity and high stocking densities. Moreover, coupled with its high growth rates, acceptable taste, low costs of production, and high profitability, this fish species is, therefore, of great economic importance in biosaline aquaponics [41, 42].

The aim of our study, therefore, was to investigate; (i) the effect of irrigating saline aquaculture wastewater on the growth, yield, and forage quality of barley cultivated under an IAAS and (ii) to elucidate the effect of salinity on the growth performance and health status of striped catfish (*Pangasianodon hypophthalmus*) reared under an IAAS.

## Materials and methods

### Plant material and experimental design

**Fig. 1:**
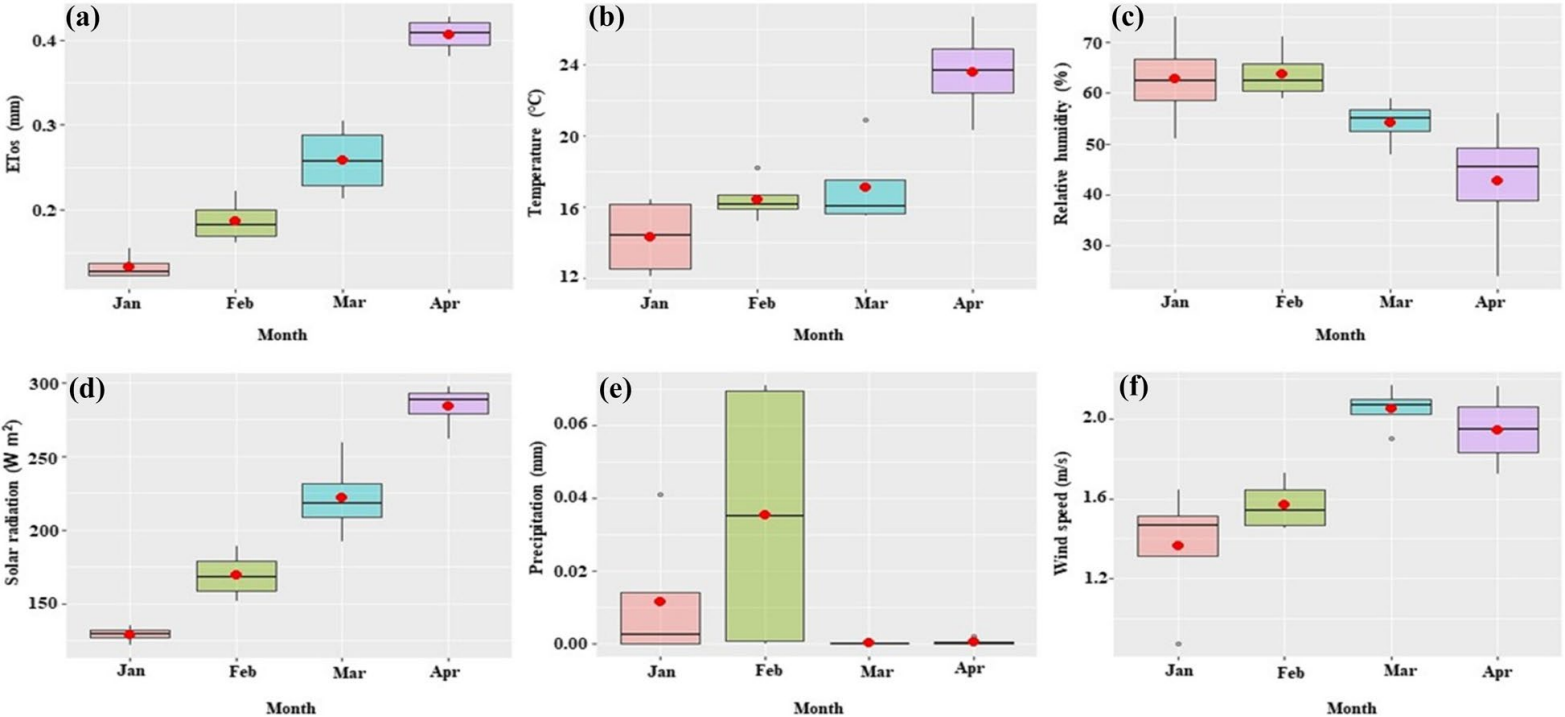
Average weather parameters recorded during the experimental period. **a**: average evapotranspiration, (**b**): average maximum temperature, (**c**): Average relative humidity, (**d**): average solar radiation, (**e**): average precipitation, and (**f**): average wind speed. Red dots indicate the mean of the climatic parameter

Barley seeds were obtained from the Agricultural Research Center (ARC) in Giza, Egypt. A field experiment was conducted between January and April 2022 at the Center for Applied Research on the Environment and Sustainability (CARES), The American University in Cairo, New Cairo Egypt (30° 01′ 11.7″ N31°29′ 59.8″ E). The experiment followed a randomized completely block design of three treatments with three replicates namely; T0: Control (freshwater mixed with chemical fertilizers), saline aquaculture wastewater treatments T1, T2, and T3 of concentrations 5,000 ppm, 10,000 ppm, and 15,000 ppm respectively. Tables 1 and 2 show the soil's chemical and physical properties as well as the chemical constituents of the salt used in this study respectively. Figure 1 shows the average weather data parameters recorded during the experimental season.

**Table 1:** Chemical and physical properties of the soil used in the experimental study

			Anions				Cations			
pH	EC (ppm)	SP	CO_3_^_^ (meq L^−1^)	HCO_3_^_^ (meq L^−1^)	Cl^−^ (meq L^−1^)	SO_4_^_^ (meq L^−1^)	Ca^++^ (meq L^−1^)	Mg^++^ (meq L^−1^)	Na^+^ (meq L^−1^)	K^+^ (meq L^−1^)
7.6	1376	23.00	-	2.36	8.47	11.61	10.61	6.28	5.13	0.43
			**Available macro and micronutrients (mg kg**^**−1**^**)**							
			N	P	K	Mn	Zn	Fe	Cu	
			47.00	14.54	48.00	0.43	0.16	1.44	0.06	

*EC* Electroconductivity, *SP* Saturation point, *CO*_*3*_^*_*^ Carbonate, *HCO*_*3*_^*_*^ Hydrogen carbonate, *Cl*^*−*^ Chloride, *SO*_*4*_^*_*^ sulfate, *Ca*^*++*^ Calcium ions, *Mg*^*++*^ Magnesium ions, *Na*^*+*^ Sodium ions, *K*^*+*^ Potassium ions, *N* Nitrogen, *P* Phosphorus, *K* Potassium, *Mn* Manganese, *Zn* Zinc, *Fe* Iron; *Cu* Copper

**Table 2:** Chemical properties of the salt used to prepare different water salinities in the experimental study

NaCl	98.50%
HCO_3_^_^	4 × 10^–3^%
SO_4_^2_^	0.31%
AsO_4_^3_^	2 × 10^–5^%
KI	5.3 × 10^–3^%
Mg	0.07%
Ca	0.07%
Cu	2 × 10^–6^%
Fe	3 × 10^–6^%
Hg	5 × 10^–6^%
Pb	2 × 10^–5^%
Cd	8 × 10^–7^%
K	0.02%
Soluble matter	0.57%
Insoluble matter	0.02%
Moisture content	0.23%

*NaCl* Sodium chloride, *HCO*_*3*_^_^ Bicarbonate, *SO*_*4*_^*2_*^ Sulfate, *AsO*_*4*_^*3_*^ Arsenate, *KI* Potassium iodide, *Mg* Magnesium, *Ca* Calcium, *Cu* Copper, *Fe* Iron, *Hg* Mercury, *Pb* Lead, *Cd* Cadmium, *K* Potassium

### Agronomical practices

Seeds were hand-sewn in rows with inter and intra-row spacing of 30 cm and 50 cm respectively based on a planting density of 1,466,667 plants/ha (three replicates per experimental unit/treatment measuring 4 m × 3.5 m). Weeding was done by hand, two weeks after sowing, and plants were drip irrigated according to crop water requirements. Insect pest and disease management were conducted according to the recommendations of the Egyptian Ministry of Agriculture. Likewise, the application of chemical fertilizers in the control treatment (fertigation) for the barley was performed according to the recommendations of the Egyptian Ministry of Agriculture.

### Agro-morphological parameter measurements

A total of six plants within the border per replicate were randomly tagged for data collection. At each data collection time point (30, 60, and 90 days after sowing (DAS)), plant heights were measured from the crown to the terminal growing point of the plant using a meter rule. Stalk diameters were measured from the second internode, bottom-up of the plant using digital vernier calipers, and averages were calculated. Leaf number and number of internodes per plant were obtained by counting healthy leaves and internodes respectively and averages were calculated. Leaf area was calculated as shown below according to the formulae of Elsahookie and Cheyed [43].

$$\mathrm{Leaf\,area }=\mathrm{ L}*\mathrm{W}*\mathrm{C}$$

where L is the leaf length, W is the leaf width, and C is the constant (0.75).

The leaf area index (LAI) was calculated according to the formulae below

$$\mathrm{LAI }=\mathrm{ leaf\,area}/\mathrm{plot\,area}$$

Chlorophyll content was measured in the early morning before mid-day using an MC-100 chlorophyll meter from Apogee Instruments, Inc, Utah, USA and data was expressed as SPAD averages. For the determination of fresh weights, forage biomass (stalks, leaves, and panicles) per six hills per replicate were harvested at the end of the experiment (soft dough stage) and weighed to obtain the fresh forage weights. The forage biomass was then bagged in paper bags, labeled, and oven-dried to a constant weight at 70 °C for 72 h, and data were expressed as g/plant.

### Fiber fraction, nutrient composition, and in vitro digestibility of forage biomass

Six plants per replicate from each treatment were pooled and samples were taken for forage quality analysis at the Regional Center for Food and Feed, Giza, Egypt. Forage biomass samples (stalks, leaves, and panicles) were ground using a Willey mill, and the fine powder passed through a 1 mm screen. Neutral detergent fiber (NDF, AOAC no. 2002.04), Acid detergent fiber (ADF, AOAC no. 973.18), and Acid detergent lignin (ADL, AOAC no. 973.18) were sequentially determined by semiautomatic ANKOM220 Fiber Analyzer (ANKOM Technology, Macedon, NY, USA). Cellulose (ADF-ADL), Hemicellulose (NDF-ADF), and Lignin were calculated from the organic matter of the detergent fiber fractions respectively. The total nitrogen content of the samples was determined by the Kjeldahl technique followed by the determination of concentrations of crude protein (CP) according to the Association of Official Analytical Chemists 2016 (AOAC no.984.13 and no. 968.06 respectively). Crude fat (CF), fiber, ash, and humidity contents were determined according to the approved methods given in A.O.A.C [44].

To assess the in vitro digestibility of the samples, the Menke and Steingass [45] gas production technique was used. Briefly, ammonium-free rumen fluid was collected in equal proportions from two animal donors (sheep) before their morning feed and put into thermo flasks. The rumen fluid was later filtered through a 1 mm sieve and the obtained filtrate was incubated at 39 °C. Rumen 27 liquor and buffer solution were mixed in a ratio of 1:2 (v/v) and all laboratory procedures for handling rumen liquor were conducted under a continuous flow of carbon dioxide gas. 200 mg test samples were fed into 100 ml capacity graduated plastic syringes and the lubricated pistons were inserted onto the syringes. 30 ml of rumen liquor (inoculum) was introduced into the plastic syringes via silicon tubes at the tips of the syringes and these were subjected to incubation (± 39 °C). Gas production was measured at 2, 4, 6, 8, 10, 12, 14, 16, 18, 20, 22, and 24 h. This experiment was conducted in triplicates. Digestible organic matter (DOM), metabolic energy (ME), and net energy (NE) were calculated as described by Menke and Steingass [45]. Total digestible nutrients (TDN) were calculated from ME values as per the equation of NRC [46]. Microbial protein (MP) was calculated as described by Czerkawski [47] whereas short-chain fatty acids (SCFA) were calculated as described by Getachew et al. [48].

### Fish growth performance and hematological parameters

Striped catfish (*Pangasianodon hypophthalmus*) were stocked in aquaculture tanks (capacity 900 L) at a stocking density of 81 fish per tank (initial weight ~ 137.0 g; initial length 24.3 cm). The fish were fed 2 – 3 times daily with commercial floating pellets supplied by Skretting Egypt. The pellets contained 28% crude protein, 5% crude lipid, 6% crude fiber, 13% ash, and 9% moisture. The feeding pattern and frequency were according to the fish biomass percentage of 2 – 3% depending on the growth and satiation. Fish growth performance parameters such as feed intake (FI), body weight gain (BWG), feed conversion ratio (FCR), specific growth rate (SGR), condition factor, and survival were calculated according to the formula below.

$$\begin{array}{l}\text{FI}\ = \text{Total amount of feed given to the fish daily/total number of fish in the tank}\\ \text{BWG}\ = \text{Final body weight} - \text{initial body weight}\\ \text{FCR}\ = \text{FI / BWG}\\ \text{SGR}\ = \text{(In (final body weight) - In (Initial body weight)) / Experimental duration}\\ \text{Condition factor}\ = \text{(final body weight / (fish body length * 3)) *100}\\ \text{Survival}\ = \text{(number of fish at the end of the experiment/number of fish at the beginning of the experiment) * 100}\end{array}$$

For hematological parameters, a group of 3 fish per salinity treatment was randomly collected and a 1 cc sterile syringe was used to draw blood from the mid-ventral line behind the anal fin and collected in purple top EDTA blood collection tubes. Blood was immediately taken to the lab for a complete blood count (CBC) test. 150 µl of blood per treatment was used for determining the CBC using the human automatic hematology analyzer (XP-300, Sysmex corporation).

### Aquaculture wastewater analysis

Aquaculture wastewater parameters such as pH, dissolved oxygen, and temperature were closely monitored using automated digital Nilebot technologies by Conative Labs to ensure optimum growth conditions for the fish. Water samples were collected every two weeks and immediately taken to the lab for quantification of ammonia, nitrites, and nitrates concentrations. Briefly, 100 ml of water was collected from the fish tanks before feeding and irrigation, and taken to the lab for measurement of the nitrogenous elements using a photometer along with the ammonia reagent kit (H193715- 01), nitrate reagent kit (H193728-01), and nitrite reagent kit (H193707-01) respectively from HANNA instruments. Specific absorbance of the nitrogenous elements was measured using the Aquaculture Photometer device (H183303). The device was set to display the concentrations of ammonia, ammonia–nitrogen, ammonium, nitrates, nitrate–nitrogen, nitrite, and nitrite-nitrogen in mg/L.

### Statistical analysis

Data analysis was performed using R-Statistical Programming Language (version 4.1.0). Before Analysis of variance (ANOVA) was conducted, data sets were tested for normality and equality of variances using Q-Q plots and Levene’s test respectively. ANOVA was conducted to test for significant differences (*P* < 0.05) among the treatments. Fisher’s Least Significant Difference (LSD) test was used to compare differences between treatment means when significant F values were observed at *P* < 0.05 level. Pearson correlation coefficient analysis was performed using the *rCorr* function to detect relationships between different variables.

## Experimental results

### Agro-morphological parameters

Results of the effect of different water salinities on the growth of barley at different data collection time points are presented in Table 3. At 30 days after sowing (DAS), no significant differences in plant height were noted among the treatments. For internode number per plant, T1 significantly (*P* < 0.05) recorded the lowest values compared to T0, T2, and T3. Data on stalk diameter, leaf area, and leaf area index (LAI) indicated that T0 significantly (*P* < 0.05) recorded higher values compared to other treatments. Results on leaf number indicated that T1 significantly (*P* < 0.05) recorded the lowest values for leaf number per plant compared to other treatments. No significant differences in SPAD values were noted among all the treatments.

**Table 3:** Agro-morphological parameters of barley cultivated under different salinity treatments

30 DAS							
Treatment	Plant height (cm)	Internode number plant^−1^	Stalk diameter (mm)	Leaf number plant^−1^	Leaf area (cm^2^)	LAI	SPAD
T0	57.10^a^ ± 3.08	1.94^a^ ± 0.24	6.08^a^ ± 0.65	4.67^a^ ± 0.49	40.40^a^ ± 4.71	2.88^a^ ± 0.34	383.00^a^ ± 73.30
T1	56.00^a^ ± 0.00	1.11^c^ ± 0.32	5.05^b^ ± 0.60	3.67^c^ ± 0.49	31.60^b^ ± 4.25	2.26^b^ ± 0.30	388.00^a^ ± 39.50
T2	56.00^a^ ± 0.00	1.61^b^ ± 0.50	5.10^b^ ± 0.79	4.06^b^ ± 0.54	32.60^b^ ± 5.49	2.33^b^ ± 0.39	363.00^a^ ± 71.30
T3	56.00^a^ ± 0.00	1.61^b^ ± 0.50	5.11^b^ ± 0.43	4.17^b^ ± 0.58	33.70^b^ ± 5.57	2.40^b^ ± 0.39	393.00^a^ ± 33.60
60 DAS							
T0	128.00^a^ ± 4.38	6.94^a^ ± 1.11	5.98^a^ ± 0.61	6.17^a^ ± 0.51	43.20^a^ ± 5.38	3.09^a^ ± 0.39	430.00^a^ ± 46.90
T1	116.00^b^ ± 5.00	5.94^b^ ± 0.80	5.68^ab^ ± 0.58	5.50^b^ ± 0.51	40.50^a^ ± 5.17	2.89^a^ ± 0.37	342.00^b^ ± 42.70
T2	102.00^c^ ± 3.96	5.44^b^ ± 0.86	5.21^c^ ± 0.63	5.56^b^ ± 0.62	41.30^a^ ± 4.71	2.95^a^ ± 0.34	322.00^b^ ± 36.00
T3	102.00^c^ ± 9.36	5.44^b^ ± 0.51	5.47^bc^ ± 0.60	5.61^b^ ± 0.50	38.30^a^ ± 7.11	2.74^a^ ± 0.51	335.00^b^ ± 52.70
90 DAS							
T0	130.00^a^ ± 3.55	4.67^bc^ ± 0.84	4.93^a^ ± 0.74	5.28^a^ ± 0.67	29.40^a^ ± 7.02	2.10^a^ ± 0.50	243.00^a^ ± 97.80
T1	110.00^b^ ± 8.31	4.94^ab^ ± 1.11	4.91^a^ ± 0.64	5.50^a^ ± 0.62	32.30^a^ ± 4.96	2.31^a^ ± 0.35	183.00^a^ ± 64.10
T2	104.00^c^ ± 5.70	5.33^a^ ± 0.77	4.92^a^ ± 0.54	5.67^a^ ± 0.69	30.00^a^ ± 6.56	2.14^a^ ± 0.47	208.00^a^ ± 75.20
T3	110.00^b^ ± 4.57	4.44^c^ ± 0.52	4.92^a^ ± 0.41	5.39^a^ ± 0.50	33.40^a^ ± 6.54	2.39^a^ ± 0.45	232.00^a^ ± 54.70

Data is expressed as mean ± SD (*n* = 6). Different lower superscript letters within each column indicate a significant difference between treatments (*P* < 0.05). T0: Control, T1: 5,000 ppm, T2: 10,000 ppm, T3: 15,000 ppm, *LAI* Leaf area index, *DAS* Days after sowing

At 60 DAS, T0 significantly (*P* < 0.05) recorded the highest plant height compared to other treatments. Likewise, T0 significantly (*P* < 0.05) higher values for internode number compared to T2 and T3. For stalk diameter, T2 significantly (*P* < 0.05) recorded lower values compared to T0 and T1. Data on leaf number per plant indicated that T0 significantly (*P* < 0.05) recorded higher values compared to T1, T2, and T3. However, no significant differences in leaf area and LAI. For SPAD, T0 significantly (*P* < 0.05) recorded higher values compared to other treatments.

At 90 DAS, T0 significantly (*P* < 0.05) recorded an increase in plant height by 1.5% compared to other treatments. However, no significant differences in stalk diameter, leaf number per plant, LAI, and SPAD were noted among all the treatments.

### Forage yield

**Fig. 2:**
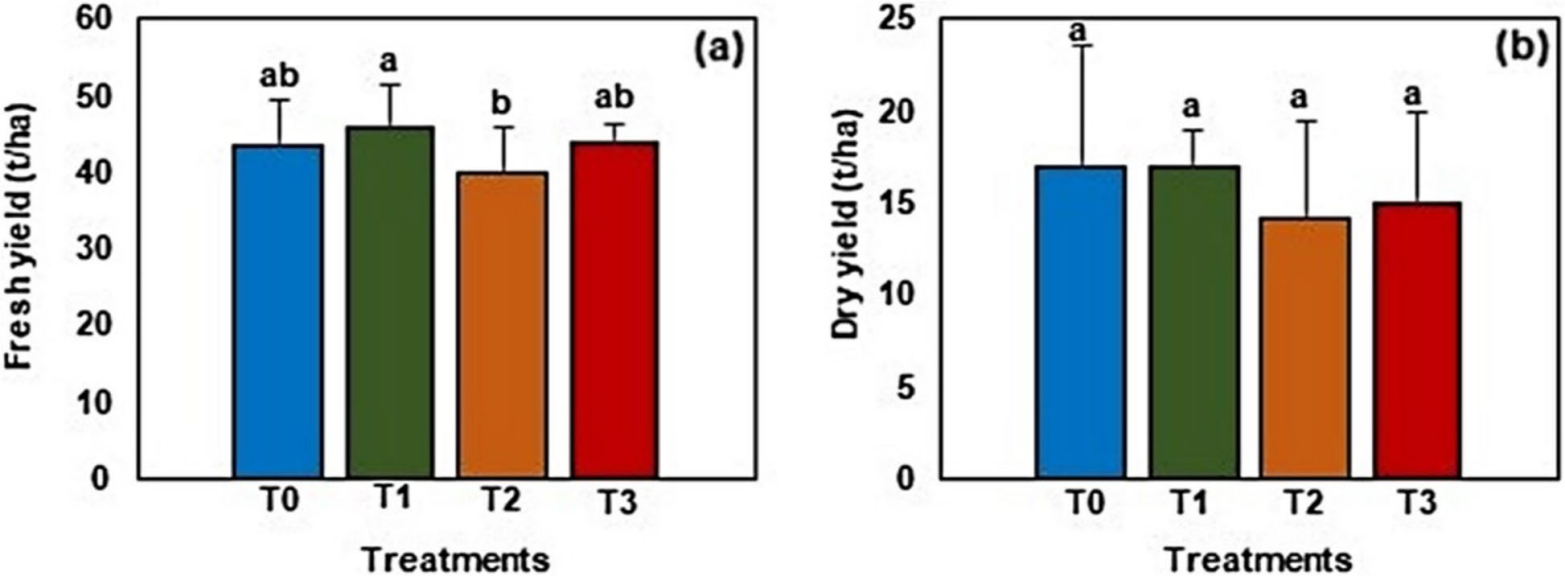
**a** Fresh and (**b**) dry yield of barley cultivated under different salinity treatments. Data is presented as mean ± SD. Error bars represent the standard deviation. Bar columns having different letters are significantly different (P < 0.05). T0: Control, T1: 5,000 ppm, T2: 10,000 ppm, T3: 15,000 ppm

Figure 2 shows the results of the forage yield of barley cultivated under different salinity treatments. The average fresh yield (Fig. 2a) ranged from 39.7 to 45.6 t/ha with T1 significantly (*P* < 0.05) recording higher values compared to T2. No significant differences in the average fresh yield were noted between T0, T1, and T3. Furthermore, the average dry yield (Fig. 2b) ranged from 14.1 to 16.9 t/ha, however, no significant differences were noted among all the treatments.

### Correlation between yield and average agro-morphological parameters

**Fig. 3:**
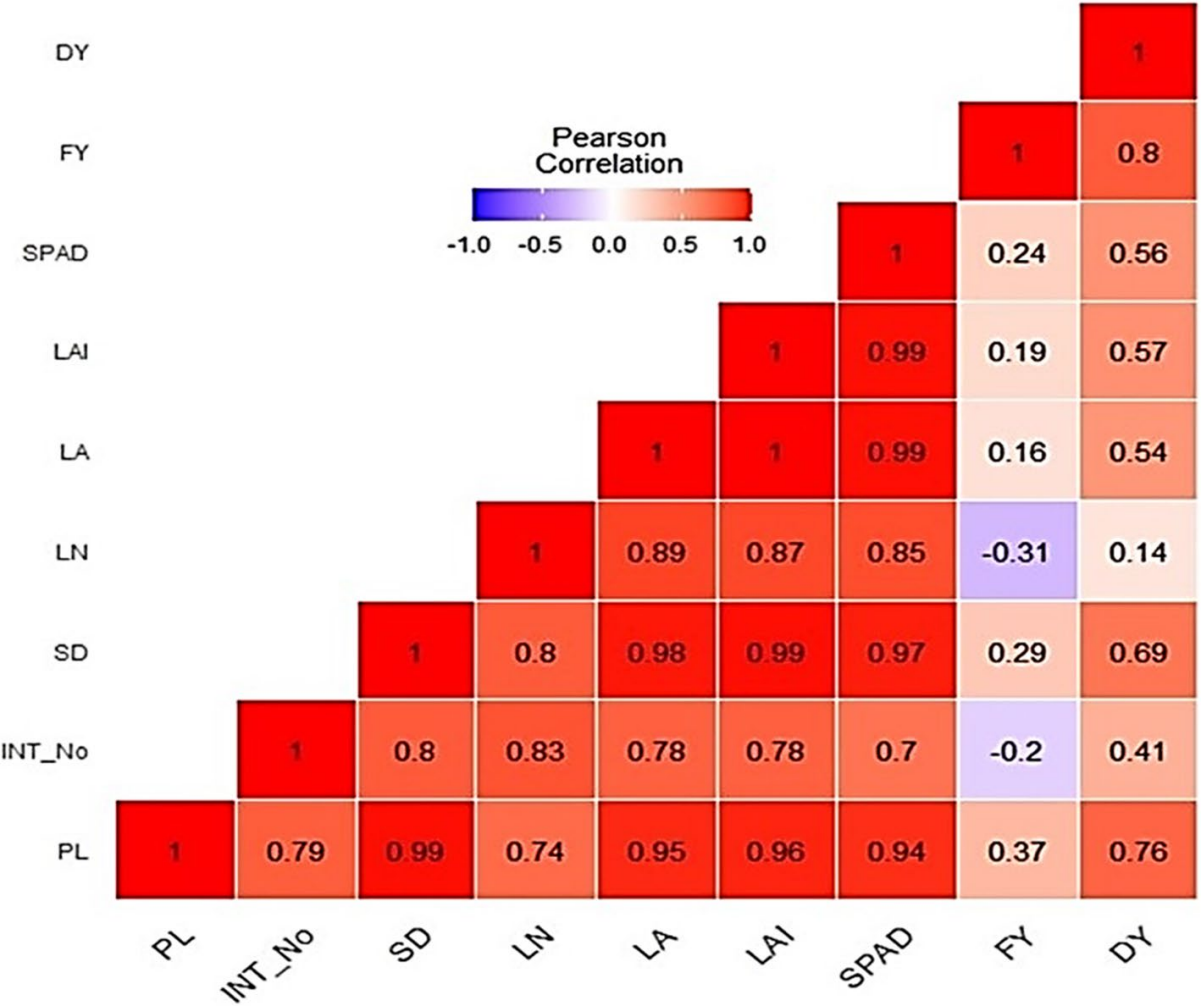
A heatmap correlation matrix of yield and average agro-morphological parameters of barley. Red and blue colors are positive and negative significant correlations, respectively, by Pearson correlation analysis. The color intensity is proportional to the correlation coefficient. PL: Plant height, INT_No: internode number, SD: Stalk Diameter, LN: Leaf Number, LA: Leaf Area, LAI: Leaf Area Index, FY: Fresh Yield, DY: Dry Yield

Figure 3 shows a heatmap of the correlation matrix yield and agro-morphological parameters of barley cultivated under different salinity treatments. There was a positive and strong correlation between dry yield and fresh yield (*r* = 0.8, *P* < 0.0001), dry yield and plant height (*r* = 0.76, *P* < 0.0001), dry yield and stalk diameter (*r* = 0.69, *P* < 0.0001). Furthermore, there was a positive and moderate correlation between dry yield and SPAD (*r* = 0.56, *P* < 0.0001), dry yield and LAI (*r* = 0.57, *P* < 0.0001), dry yield and leaf area (*r* = 0.54, *P* < 0.0001), as well as dry yield and internode number per plant (*r* = 0.41, *P* < 0.0001). However, there was a negative correlation between fresh yield and leaf number (*r* = -0.31, *P* < 0.0001) as well as fresh yield and internode number per plant (*r* = -0.2, *P* < 0.0001).

### Fiber fraction and nutrient composition of forage biomass

Fiber fraction and nutrient composition of forage biomass under different salinity treatments were evaluated and results are presented in Table 4. T0 significantly (*P* < 0.05) recorded higher values for neutral detergent fiber (NDF) compared to T1 and T3. Likewise, T0 significantly (*P* < 0.05) recorded the highest values for acid detergent fiber (ADF) compared to other treatments. For acid detergent lignin, however, T0 and T3 significantly (P < 0.05) recorded lower values compared to T1 and T2. No significant differences in the hemicellulose (HEM) and lignin (LIG) contents were noted among all the treatments. Data on cellulose (CEL) content showed that T0 and T3 significantly had the lowest values compared to T1 and T2. The crude protein (CP) content ranged from 4.40% to 7.53% with T0 significantly (*P* < 0.05) recording the highest CP content compared to other treatments. For crude fats (CF) and fiber content, T0 significantly (*P* < 0.05) recorded the lowest values compared to other treatments. The ash content percentage ranged from 6.80% to 9.57% with T0 and T1, significantly (*P* < 0.05) recording lower values compared to T2 and T3. No significant difference in the humidity percentage was noted among all the treatments.

**Table 4:** Fiber fraction and nutrient composition of barley cultivated under different salinity treatments

Treatment	NDF (%)	ADF (%)	ADL (%)	HEM (%)	CEL (%)	LIG (%)	CP (%)	CF (%)	Fiber (%)	Ash (%)	Humidity (%)
T0	55.30^a^ ± 5.27	35.20^a^ ± 1.29	3.92^b^ ± 1.06	21.82^a^ ± 2.61	23.21^c^ ± 1.11	3.60^a^ ± 0.40	7.53^a^ ± 0.56	1.80^c^ ± 0.27	25.01^c^ ± 2.10	6.80^b^ ± 0.96	4.07^a^ ± 0.56
T1	51.10^bc^ ± 1.69	29.80^c^ ± 0.90	5.05^a^ ± 0.91	21.29^a^ ± 1.21	24.78^b^ ± 0.87	3.65^a^ ± 0.42	4.40^c^ ± 0.26	2.12^b^ ± 0.23	28.83^a^ ± 0.67	7.80^b^ ± 0.85	4.40^a^ ± 0.23
T2	53.20^ab^ ± 1.87	31.40^b^ ± 0.20	5.28^a^ ± 1.19	21.76^a^ ± 1.95	26.13^a^ ± 1.13	3.57^a^ ± 0.36	4.63^c^ ± 0.43	2.34^a^ ± 0.12	28.86^a^ ± 0.59	9.57^a^ ± 1.53	4.17^a^ ± 0.35
T3	49.10^c^ ± 1.96	26.60^d^ ± 0.95	4.05^b^ ± 0.41	21.81^a^ ± 1.70	23.62^c^ ± 1.20	3.49^a^ ± 0.46	5.97^b^ ± 0.28	2.23^ab^ ± 0.10	26.76^b^ ± 1.05	9.33^a^ ± 0.89	4.10^a^ ± 0.10

Data is expressed as mean ± SD (*n* = 3). Different lower superscript letters within each column indicate a significant difference between treatments (*P* < 0.05). T0: Control, T1: 5,000 ppm, T2: 10,000 ppm, T3: 15,000 ppm, *NDF* Neutral Detergent Fiber, *ADF* Acid Detergent Fiber, *ADL* Acid Detergent Lignin, *HEM* Hemicellulose, *CEL* Cellulose, *LIG* Lignin, *CP* Crude Protein, *CF* Crude fat

### In vitro digestibility of the forage biomass

Results of the in vitro digestibility of forage biomass under different salinity treatments are presented in Table 5. The digestible organic matter (DOM) content ranged from 47% to 49.4% with T2 and T3 recording higher values compared to other treatments. Likewise, T2 and T3 recorded higher values for short-chain fatty acids (SCFA), total digestible nutrients (TDN), microbial protein (MP), and metabolic energy (ME) compared to other treatments. However, no significant differences were noted. Data on the net energy (NE) showed that T0 significantly (*P* < 0.05) had the highest values compared to T1 and T2.

**Table 5:** Results of in vitro digestibility of barley cultivated under different salinity treatments

Treatment	DOM (%)	SCFA (mmol/ml gas)	TDN (%)	MP (g/kg DOM)	ME (Mcal/kg DM)	NE (Mcal/IB)
T0	47.60^a^ ± 3.23	0.71^a^ ± 0.11	48.10^a^ ± 3.19	57.50^a^ ± 3.90	1.69^a^ ± 0.15	3.52^a^ ± 0.11
T1	47.00^a^ ± 2.71	0.74^a^ ± 0.08	48.10^a^ ± 2.26	56.70^a^ ± 3.26	1.69^a^ ± 0.10	3.38^c^ ± 0.08
T2	49.10^a^ ± 1.73	0.79^a^ ± 0.05	49.60^a^ ± 1.49	59.20^a^ ± 2.08	1.76^a^ ± 0.07	3.45^b^ ± 0.06
T3	49.40^a^ ± 0.18	0.78^a^ ± 0.01	49.80^a^ ± 0.14	59.60^a^ ± 0.21	1.76^a^ ± 0.01	3.52^ab^ ± 0.01

Data is expressed as mean ± SD (*n* = 3). Different lower superscript letters within each column indicate a significant difference between treatments (*P* < 0.05). T0: Control, T1: 5,000 ppm, T2: 10,000 ppm, T3: 15,000 ppm, *DOM* Digestible Organic Matter, *SCFA* Short-Chain Fatty Acids, *TDN* Total Digestible Nutrients, *MP* Microbial Protein, *ME* Metabolic Energy, *NE* Net Energy

### Correlation between fiber fraction, nutrient composition, and in vitro digestibility

**Fig. 4:**
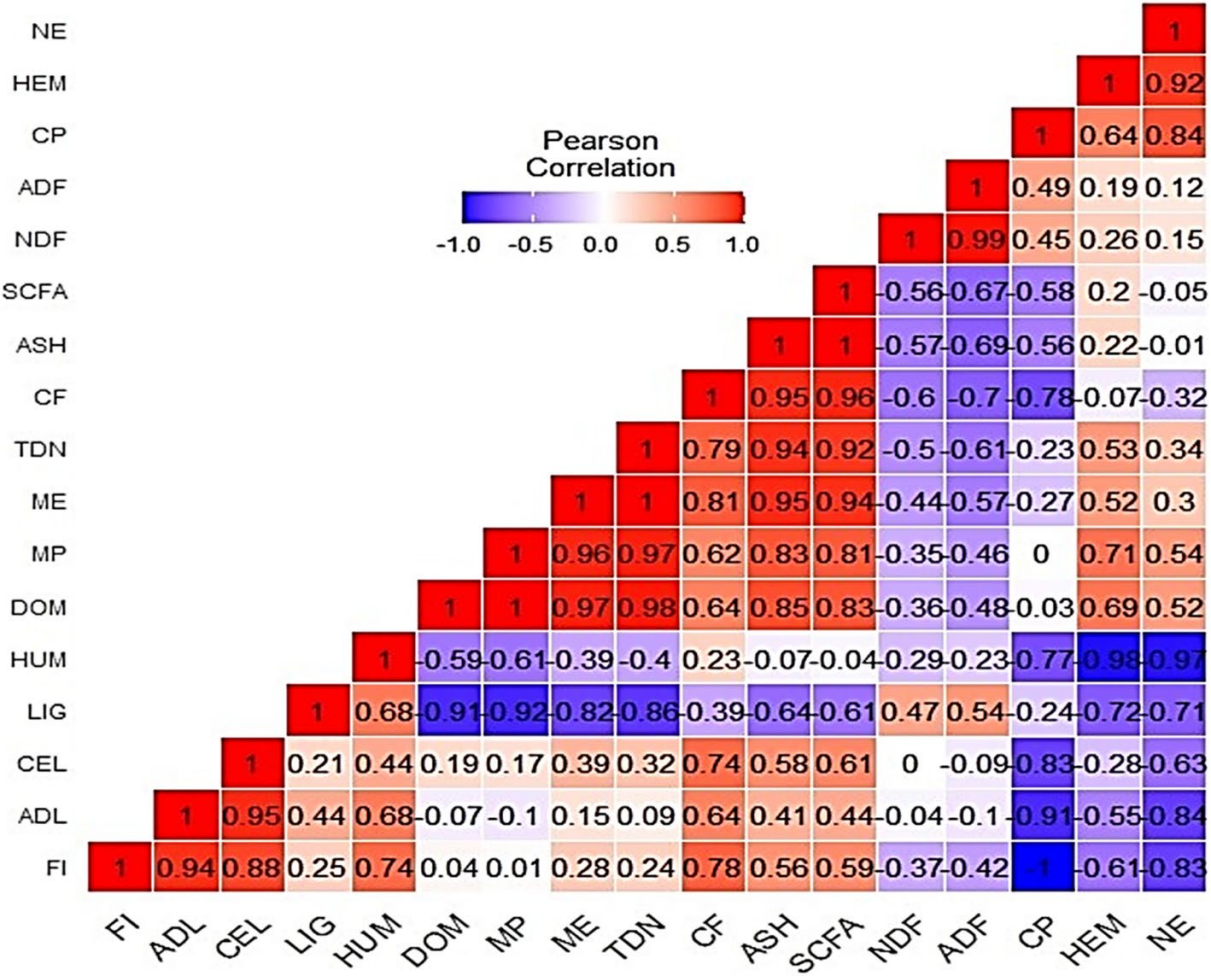
A heatmap correlation matrix of fiber fraction, nutrient composition, and in vitro digestibility of barley. Red and blue colors are positive and negative significant correlations, respectively, by Pearson correlation analysis. The color intensity is proportional to the correlation coefficient. FI: Fiber, ADL: Acid Detergent Lignin, CEL: Cellulose, LIG: Lignin, HUM: Humidity, DOM: Digestible Organic Matter, MP: Microbial Protein, ME: Metabolic Energy, TDN: Total Digestible Nutrients, CF: Crude Fat, ASH: Ash, SCFA: Short-Chain Fatty Acids, NDF: Neutral Detergent Fiber, ADF: Acid Detergent Lignin, CP: Crude Protein, HEM: Hemicellulose, NE: Net Energy

Figure 4 shows a heatmap of the correlation analysis between fiber fraction, nutrient composition, and in vitro digestibility of barley cultivated under different salinity treatments. A positive and strong correlation was noted between NE and HEM (*r* = 0.92, *P* < 0.0001), NE and CP (*r* = 0.84, *P* < 0.0001), Ash and CF (*r* = 0.95, *P* < 0.0001), ash and TDN (*r* = 0.94, *P* < 0.0001), ash and ME (*r* = 0.95, *P* < 0.0001), ash and MP (0.83, *P* < 0.0001), as well as ash and DOM (*r* = 0.83, *P* < 0.0001). Likewise, there was a positive and strong correlation between ADF and NDF (*r* = 0.99, *P* < 0.0001), as well as ADL and FI content (*r* = 0.94, *P* < 0.0001).

### Fish growth performance and hematological parameters

Results of the growth performance indices of *Pangasianodon hypophthalmus* are presented in Table 6. No significant differences were noted in the initial weights of fish across all salinity treatments. However, fish reared in T1 recorded significantly (*P* < 0.05) higher values for final weight, body weight gain (BWG), and specific growth rate (SGR) compared to those reared in T3. For the feed conversion ratio (FCR) and condition factor, fish reared in T1 exhibited better FCR and condition factor than those reared in T2 and T3 but no significant differences were noted. However, the feed intake (FI) of fish reared in T1 was significantly (*P* < 0.05) higher than those reared in T2 and T3. However, the survival percentage of fish varied across the treatments with T2 significantly (*P* < 0.05) recording higher values for survival percentage followed by T1 and T3 respectively.

**Table 6:** Growth performance of Pangasianodon hypophthalmus reared under different water salinities

Treatment	Initial weight (g)	Final weight (g)	BWG (g)	FI (g)	FCR	SGR (%)	Condition factor	Survival (%)
T1	136.00^a^ ± 0.67	354.92^a^ ± 32.13	218.43^a^ ± 31.64	317.65^a^ ± 0.00	1.48^a^ ± 0.22	1.18^a^ ± 0.11	0.85^a^ ± 0.09	97.53^b^ ± 0.00
T2	137.00^a^ ± 1.06	322.10^ab^ ± 24.81	184.64^ab^ ± 24.98	284.28^b^ ± 0.00	1.56^a^ ± 0.20	1.05^ab^ ± 0.09	0.88^a^ ± 0.08	100.00^a^ ± 0.00
T3	138.00^a^ ± 0.80	282.15^b^ ± 6.70	144.58^b^ ± 7.25	248.31^c^ ± 0.00	1.72^a^ ± 0.09	0.89^b^ ± 0.03	1.00^a^ ± 0.10	93.83^c^ ± 0.00

Data is expressed as mean ± SD (*n* = 3). Different lower superscript letters within each column indicate a significant difference between treatments (*P* < 0.05). T1: 5,000 ppm, T2: 10,000 ppm, T3: 15,000 ppm, *BWG* Body Weight Gain, *FI* Feed Intake, *FCR* Feed Conversion Ratio, *SGR* Specific Growth Rate

**Fig. 5:**
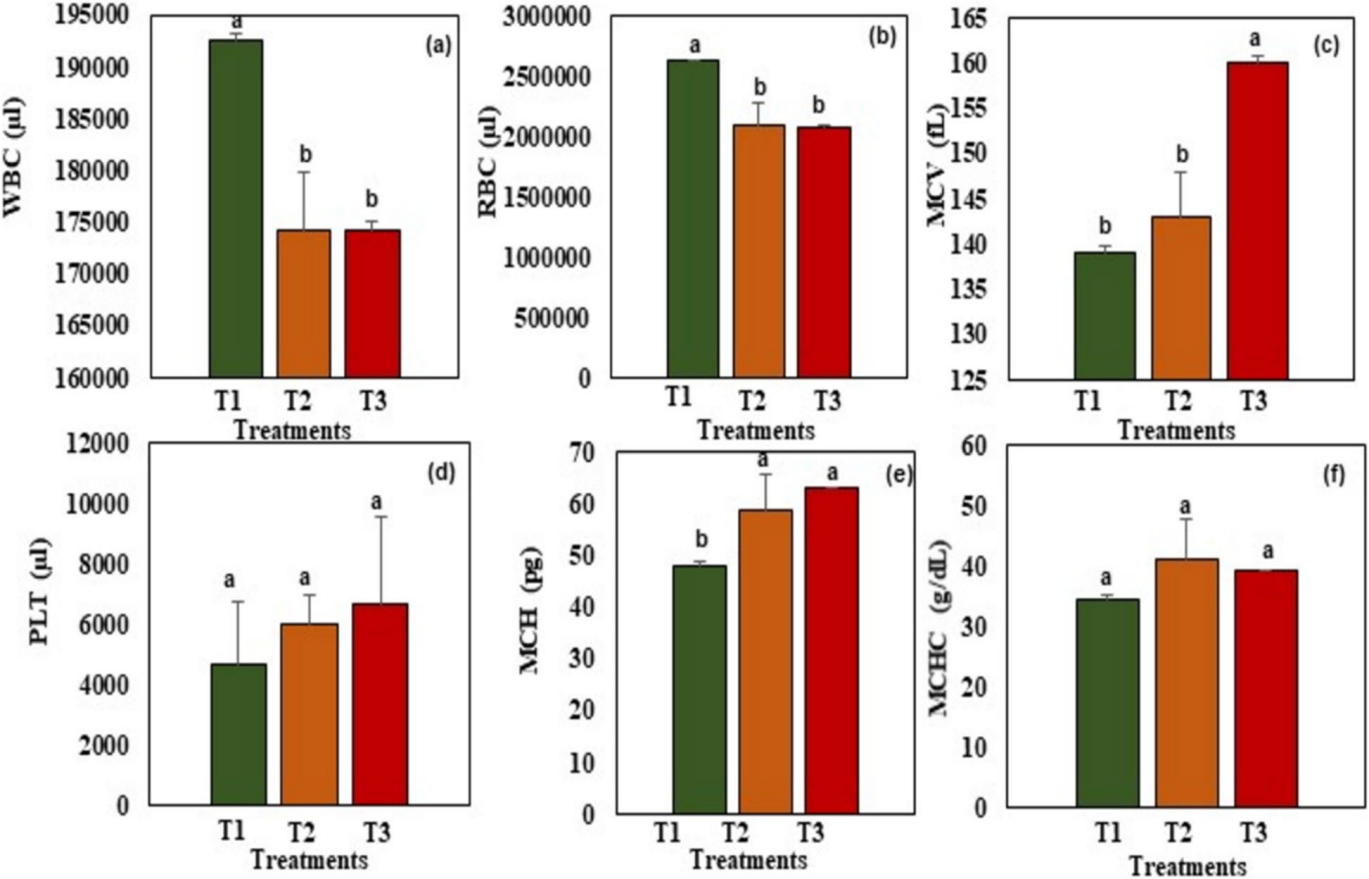
Hematological parameters of Pangasianodon hypophthalmus reared under different salinity treatments. Data is presented as mean ± SD (*n* = 3). Error bars represent the standard deviation. Bar columns have different letters are significantly different at *P* < 0.05. T1: 5,000 ppm, T2: 10,000 ppm, T3: 15,000 ppm. **a**: White blood cells (WBC), (**b**): Red blood cells (RBC), (**c**): Mean corpuscular volume (MCV), (**d**) PLT: Platelets, (**e**): Mean corpuscular hemoglobin, (**f**): Mean corpuscular hemoglobin concentration

The influence of salinity on the hematological parameters of P. *hypophthalmus* is presented in Fig. 5. Fish reared in treatment T1 significantly (*P* < 0.05) had the highest white blood cell (WBC) (Fig. 5a) and red blood cell (RBC) (Fig. 5b) concentration compared to those reared in T2 and T3. However, fish reared in treatment T1 significantly (*P* < 0.05) recorded lower values for mean corpuscular volume (MCV) (Fig. 5c), and mean corpuscular hemoglobin (MCH) (Fig. 5e) compared to those reared in treatments T2 and T3. No significant differences in the concentration of platelets (PLT) (Fig. 5d) and mean corpuscular hemoglobin concentration (MCHC) (Fig. 5f) was noted among all treatments.

### Aquaculture wastewater quality

Table 7 summarizes the results of the aquaculture wastewater quality. The results showed no significant differences in the concentration of ammonia (NH_3_), ammonium (NH_4_^+^), and ammonia–nitrogen (NH_3_ – N) among all the treatments. Although T3 recorded higher values for concentration of nitrite (NO_2-_), nitrite-nitrogen (NO_2-_ – N), nitrate (NO_3-_), and nitrate-nitrogen (NO_3-_ – N), no significant differences were noted among T3, T2, and T1.

**Table 7:** Results of the aquaculture wastewater quality at different salinity treatments

Treatment	Ammonia (mg/L)	Ammonium (mg/L)	Ammonia–Nitrogen (mg/L)	Nitrite (mg/L)	Nitrite-Nitrogen (mg/L)	Nitrate (mg/L)	Nitrate-Nitrogen (mg/L)
T1	2.69^a^ ± 2.54	2.98^a^ ± 2.69	2.21^a^ ± 2.09	13.30^a^ ± 11.50	4.33^a^ ± 3.51	60.10^a^ ± 60.20	13.60^a^ ± 13.60
T2	4.87^a^ ± 4.78	5.17^a^ ± 5.07	4.00^a^ ± 3.93	19.00^a^ ± 16.00	6.00^a^ ± 5.00	69.70^a^ ± 63.30	15.60^a^ ± 14.50
T3	2.78^a^ ± 2.41	2.93^a^ ± 2.55	2.27^a^ ± 1.98	20.70^a^ ± 14.60	7.33^a^ ± 4.73	87.70^a^ ± 45.30	19.80^a^ ± 10.20

Data is expressed as mean ± SD (*n* = 3). Different lower superscript letters within each column indicate a significant difference between treatments (*P* < 0.05). T1: 5,000 ppm, T2: 10,000 ppm, T3: 15,000 ppm

## Discussion

Freshwater scarcity is one of the major challenges facing the Egyptian agricultural sector today hence the utilization of brackish water for the production of salt-tolerant plants and fish is one of the potential solutions to safeguard the country’s food security. In the current study, irrigating plants with saline aquaculture wastewater of different salinities did not severely affect the forage yield of barley (Fig. 2). It is worth noting that barley is one of the most commonly studied model crops on the inheritance and mechanisms of salinity tolerance due to its ability to tolerate salinity levels reaching up to 250 mM NaCl (equivalent to 40% sea water) [39, 40]. For instance, Mwando et al. [49] investigated the genome-wide association of salinity tolerance in barley during germination. Using approximately 24,000 genetic markers to detect marker-trait associations (MTA) and their associated genes for salinity tolerance during germination, the authors detected 19 quantitative trait loci (QTLs) containing 52 significant salt-tolerance-associated markers across all chromosomes and 4 genes belonging to 4 family functions (*Piriformospora indica*-insensitive protein 2, Protein kinase superfamily protein, Lipase 1, and Heat shock protein 21) underlying the predicted MTAs. In another study, Zhou et al. [50] conducted a glasshouse experiment to identify QTLs associated with salinity stress tolerance in 172 doubled-haploid lines generated from a salt-tolerant barley genotype, YYXT during the vegetative growth phase. The authors identified 5 QTLs for salinity tolerance on chromosomes 1H, 2H, 5H, 6H, and 7H which accounted for more than 50% of the phenotypic variation. Similarly, Xue et al. [51] conducted a study to identify the QTLs associated with salinity stress tolerance in barley during its late growth stage and detected 13 QTLs under salinity stress. Moreover, major QTLs controlling tiller number, spikes per line, and spikes per plant were mapped on the same region (i.e. a region flanked by the markers bPb-1278 and bPb-8437) on chromosome 4H and were highly expressed under salinity stress hence indicating the significance of chromosome 4H in salinity tolerance in barley.

Although our results did not show a severe negative impact of salinity on the forage yield of barley, we observed a non-significant decline in certain agro-morphological parameters such as stalk diameter, leaf area, leaf area index (LAI), and chlorophyll content (SPAD) (Table 3). These observations could be attributed to several factors such as ionic and oxidative stress that lead to the production and release of reactive oxygen species (ROS) which cause chlorophyll degradation [52–54] and lipid peroxidation of cell membranes [53, 55, 56]. In a study conducted on several genotypes of barley (*Hordeum vulgare*), Akhter et al. [57] reported that changes in the length of plant shoots, roots, and photosynthetic activity were positively correlated under salinity stress, concluding that salinity stress disrupted the chlorophyll molecules and proteins in photosystem II (PSII) which resulted in the disturbance in electron transfer between donor and acceptor sites in PSII mostly in the salinity sensitive genotype (B-14011). In another study, Shahzad et al. [58] observed that irrigating maize (*Zea mays*) with saline water (8000 ppm) negatively impacted the photosynthetic activity of plants as indicated by a decline in the chlorophyll content of plant leaves and growth. Salinity stress has also been reported to induce changes in the anatomy of several plant species as a result of changes in turgor potential, osmotic potential, and a decline in nutrient uptake [57, 59, 60]. For instance, Abrar et al. [61] investigated the impact of salinity on the agro-morphological traits of *Jatropha curcas* and observed a significant reduction in plant height and stem diameter under salinity stress. In young citrus rootstocks, Othman et al. [59] observed a reduction in nutrient uptake in sour orange (*Citrus aurantium*) irrigated with saline water reaching up to 12,000 ppm which led to a decline in several agro-morphological traits such as stem diameter, plant height, and leaf area. Similarly, Kheloufi & Mansouri [60] reported anatomical changes in root and stem tissues of *Acacia karroo* and *Acacia saligna* cultivated under highly saline conditions.

Positive and strong correlations were noted between dry weight and stalk diameter, plant height, SPAD, as well as fresh weight (Fig. 3). This is because variations in the aforementioned agro-morphological parameters are anticipated to be the result of changes in the moisture content of plant tissues and temperature which have a direct effect on the dry weight [62]. During the experimental period of our study, there was an increase in temperature and solar radiation (Fig. 1b and d) which we anticipate could have led to a decline in the moisture content of plant tissues thus causing an impact on the variation of the studied agro-morphological parameters regardless of the salinity treatment. Solar radiation has been previously reported to cause changes in atmospheric temperatures [63–65] which could also impact plant growth, and depending on the plant species, high solar radiation can improve or reduce plant growth and yield (i.e. in terms of fresh and dry weights). For instance, Simonneau et al. [66] observed changes in stem diameters of peach trees (*Prunus persica* (L.) Batsch cv ‘Maycrest’), and this was closely related to solar radiation and moisture content of plant tissues. However, Suzuki et al. [67] have recently shown that inducing solar radiation stress on sweet potato (*Ipomoea batatas* (L.) Lam) grown in a multilayer cultivation system led to an increase in the number of leaves in the lower layer. Furthermore, the authors reported an increase in the amount of dry biomass of sweet potatoes as solar radiation increased.

Our study also evaluated the influence of different water salinities on the forage quality of barley (*Hordeum vulgare***)** cultivated under an integrated aquaculture-agriculture system (IAAS) (Tables 4 and 5). The amount of ash in the forage biomass was affected by salinity with higher values recorded in highly saline conditions (T2: 10,000 ppm and T3: 15,000 ppm). Our results are in agreement with previous studies on *Panicum maximum* [68], *Sorghum bicolor* [69], *Bassia scoparia* [70], *Pennisetum glaucum* (L.) [71], *Melilotus albus* and *Medicago sativa* (L.) [72]. Ash is indeed a representative of the concentration of nutrients in plant tissues and thus a potential index of forage quality [69]. This is because, under saline conditions, plants absorb salts and translocate them to the shoots leading to an increased accumulation of salts in plant tissues [69, 70, 72]. Neutral detergent fiber (NDF) and acid detergent fiber (ADF) are one of the most crucial factors used to assess forage quality and as such, forages with lower NDF and ADF facilitate more dry matter intake in ruminants [69, 73]. Salinity stress reduced the NDF and ADF of plants with the lowest values recorded in highly saline conditions (T3). Our results are similar to those reported on *Lolium multiflorum*, Lam. by Ben-Ghedalia [74], *Cynodon dactylon,* and *Pennisetum clandestinum* by Robinson et al. [75]. However, Hedayati-Firoozabadi et al. [69] observed reduced forage quality in terms of increased NDF and ADF of *Sorghum bicolor* intercropped with *Bassia indica* under highly saline conditions. The difference in results could be attributed to differences in plant species, harvest stage, and experimental conditions. Although plants cultivated under saline conditions exhibited lower values for NDF and ADF, their crude protein (CP) content was lower than that of the control. This could be due to the accumulation of salts in the root zone which caused damage to the root system and hence impeded the absorption of nutrients from the soil [76]. For good quality forages, the CP content should be ≥ 7.0 as recommended by Milford & Minson [77]. The high CP content of plants in the control could be attributed to higher concentrations of nitrogen in the inorganic fertilizer compared to that in the saline aquaculture wastewater. Previous studies have shown that application of nitrogen fertilizers improves the CP content of forages [78–81]. CP is the most important nutrient for ruminants as it facilitates the activity of rumen microbes involved in milk production and the maintenance of meat quality [82, 83]. Rumen fermentation as represented by digestible organic matter (DOM), microbial protein (MP), short-chain fatty acids (SCFA), total digestible nutrients (TDN), metabolic energy (ME), and net energy (NE) indicated no significant differences among treatments except for NE. Moreover, there was a strong and positive correlation between NE and hemicellulose (HEM) as well as NE and CP (Fig. 4). NE is an index that is used to assess the usefulness of silage in the nutrition of dairy animals [84]. As a rule of thumb, the higher the NE, the better the silage quality of the forage. Hence based on the results of our studies, barley cultivated under highly saline conditions (15,000 ppm) and harvested at the soft dough stage could still possess good silage properties. Note that barley is one of the most salt-tolerant crops and hence a model plant for studies on the mechanisms of salinity tolerance in grain crops [40, 85].

The influence of salinity on the growth performance (Table 6) and hematological parameters (Fig. 5) of *Pangasianodon hypophthalmus* were assessed. There were significant differences in the fish growth performance parameters such as the feed intake (FI), final weight (FW), body weight gain (BWG), specific growth rate (SGR), and survival percentage among the treatments. Fish reared in highly saline conditions (T2 and T3) exhibited lower FI due to salinity stress and this is in agreement with previous studies on *Oreochromis* sp. [86, 87], *Cyprinus carpio* [88, 89], *Ctenopharyngodon idella* [90, 91], and juvenile *P. hypophthalmus* [41]. It is worth noting that reduced FI in freshwater fish is crucial to relieve high osmotic stress under highly saline conditions [90, 91] which could explain the reduced FI of *P*. *hypophthalmus* in our study. Likewise, a decline in FW, BWG, and SGR mostly in extremely saline conditions (T3; 15,000 ppm) is attributed to increased catabolism of lipids and carbohydrates in fish to produce more energy required for enhancing the fish’s tolerance to salinity stress [92]. In the same regard, the survival percentage of fish reared in T1 was significantly lower compared to that of fish reared in T2 and T3 and we suggest that the observed result could be due to management practices rather than salinity. For instance, high stocking densities lead to increased oxygen demand in fish [93–95], and failure to maintain the oxygen levels at the desired concentrations of the organism could result in death.

Overall, *P*. *hypophthalmus* showed tolerance to salinity stress up to 10,000 ppm without significant detrimental effects on growth performance in terms of BWG, FCR, SGR, and condition factor. Our results are in agreement with previous studies on the growth performance of *P. hypophthalmus* under different salinity regimes [17, 41, 96, 97]. For example, Nguyen et al. [96] assessed the effect of sublethal salinities (2, 6, 10, 14, and 18 g/L) on the stress response and growth performance of *P. hypophthalmus* juveniles and observed good survival and growth performance (weight gain (WG), daily weight gain (DWG), SGR, and FCR) of fish at water salinities from 2 to 10 g/L. Fish reared in higher water salinities (14 and 18 g/L) exhibited an increased accumulation of blood glucose and cortisol which indicated stress thus leading to poor growth performance. Likewise, Jahan et al. [17] investigated the growth response of *P. hypophthalmus* reared under 0, 4, 8, and 12% salinity levels and observed that salinity levels from freshwater to 8% (8000 ppm) showed optimal conditions in terms of fish survival and growth performance parameters such as WG and SGR, with salinity level 4% (4000 ppm) showing the best FCR. Similarly, Abdel-Latif et al. [41] observed no adverse effects on the growth of *P. hypophthalmus* reared at 4% salinity levels. Moreover, the authors suggested that this fish species can tolerate salinity levels reaching up to 8% as indicated by a good FI and survival. In another study, Ha et al. [97] observed an increasing trend in the growth of *P. hypophthalmus* reared in water salinities ranging from 0 to 9%.

Just like in mammals, fish blood cells such as white blood cells (WBC) and red blood cells (RBC) are important hematological parameters used to assess fish health [98, 99]. According to the results of our study, there was a significant decline in the concentration of WBC and RBC in fish reared in T2 and T3 compared to T1 (Fig. 5a and b) hence indicating an impairment in fish health under highly saline conditions. Moreover, an increase in the mean corpuscular volume (MCV), mean corpuscular hemoglobin (MCH), and platelet concentration further attests to the health impairment of fish reared under extremely saline conditions and this is in agreement with some of the previous studies [100–102]. However, other studies have slightly shown contradictory results [103, 104] and this could be attributed to differences in fish species, initial body weight, type of feed, and experimental conditions. Overall, this study suggests that *P*. *hypophthalmus* can be reared in saline conditions not exceeding 10,000 ppm without severely affecting the health status of fish.

Accumulation of nitrogenous elements such as ammonia in rearing tanks is an important indicator of a decline in water quality in aquaculture. In our study, the T2 salinity treatment significantly had higher levels of ammonia (NH_3_), ammonium (NH_4_^+^), and ammonia–nitrogen (NH_3_ – N) accumulation compared to T1 and T3 as shown in \* MERGEFORMAT Table 7. This is because exposing fish to high water salinities leads to increased amino acid catabolism which results in the accumulation of ammonia in water [105]. Moreover, as water salinity increases, so does the equilibrium constant (pK) of ammonia thus leading to its excretion and accumulation in aquatic ecosystems [105, 106]. However, the T3 salinity treatment exhibited a decline in the accumulation of NH_3_, NH_4_^+^, and NH_3_ – N despite fish being exposed to extremely saline conditions (15,000 ppm). This is because, at extremely high salinities, the metabolism of fish changes with more preference given to lipids and carbohydrates as substrates for energy production that would aid in building up tolerance to salinity stress [92]. It is also interesting to note that the concentration of nitrates (NO_3-_), and nitrate-nitrogen (NO_3-_ – N) varied across the salinity treatments although no significant differences were noted. This could explain why there were no significant differences in agro-morphological parameters (such as stalk diameter, leaf number, leaf area index (LAI), and chlorophyll content (SPAD)) and dry yield of plants.

## Conclusion

In conclusion, therefore, the current study revealed that irrigating barley with saline aquaculture wastewater at different salinities (5000 ppm, 10, 000 ppm, and 15,000 ppm) does not severely impact the forage yield and forage quality of the crop. Furthermore, rearing striped catfish (*Pangasianodon hypophthalmus*) in water salinities not exceeding 10,000 ppm does not severely affect the growth performance and health status of the fish. Hence, the integration of *P*. *hypophthalmus* and barley production in water salinities below 15,000 ppm could be a feasible alternative for safeguarding food and feed security in regions affected by freshwater scarcity. Moreover, the salinity regime of 5,000 ppm could bring higher economic gains to farmers regarding higher crop yields (fish and forage yield).

## Data Availability

The datasets generated during and/or analyzed during the current study are available from the corresponding author upon reasonable request.
